# Virtual reality-based cognitive training in mild cognitive impairment: a systematic review

**DOI:** 10.3389/fnagi.2026.1784911

**Published:** 2026-03-11

**Authors:** Giorgia Francesca Scaramuzzi, Valerio Manippa, Klaudia Helena Pawlik, Myrto Gkanatsa, Ester Cornacchia, Daphne Gasparre, Aurora Bonvino, Giulio Lancioni, Paolo Taurisano

**Affiliations:** 1 Department of Translational Biomedicine and Neurosciences, University of Bari Aldo Moro, Bari, Italy; 2 Department of Precision and Regenerative Medicine and Ionian Area, University of Bari Aldo Moro, Bari, Italy; 3 Interdisciplinary Department of Medicine, University of Bari Aldo Moro, Bari, Italy; 4 Lega F. D’Oro Research Center, Osimo, Osimo, Italy

**Keywords:** cognitive stimulation, dementia, digital rehabilitation, innovative treatments, neurodegenerative disorders

## Abstract

**Introduction:**

Mild cognitive impairment (MCI) is recognized as a transitional phase preceding dementia and represents a crucial window for intervention. Conventional cognitive training (CCT) often lacks engagement and sustained participation, limiting its effectiveness in slowing cognitive decline. Virtual reality-based cognitive training (VRCT) has emerged as a promising alternative, offering immersive and interactive environments that may enhance cognitive training outcomes in older adults.

**Methods:**

This systematic review aims to compare the effectiveness of VRCT with CCT in individuals with MCI. A systematic search was conducted using Web of Science and PubMed, yielding 2,256 initial records. After removal of duplicates and application of eligibility criteria, seven randomized controlled trials (RCTs) were included. Key outcomes assessed included the DSM cognitive domain, general cognition, functional autonomies, and psychoaffective status. Risk of bias was evaluated using the Cochrane RoB 2 tool.

**Results:**

VRCT demonstrated enhanced effectiveness compared to conventional interventions in improving cognitive domains, and in particular general cognition, complex attention, and perceptual-motor function compared with CCT.

**Discussion:**

VRCT shows promise as an engaging and potentially effective intervention for individuals with MCI. Despite encouraging results, further high-quality, multicenter trials with standardized outcome measures and long-term follow-up are needed to establish its clinical utility and generalizability.

## 1 Introduction

In recent decades, improvements in healthcare, nutrition, and living standards have substantially increased life expectancy (Van Leeuwen et al., 2019). Although this demographic shift reflects major health achievements, it has also been associated with a growing prevalence of age-related diseases, particularly those affecting cognitive functions (Murman, 2015; Salari et al., 2025). The global burden of cognitive decline and dementia is projected to rise sharply in the coming decades, posing major clinical, economic, and societal challenges (Nichols et al., 2022; Alzheimer’s Association, 2024). As a result, identifying effective strategies to prevent or delay cognitive deterioration in aging populations has become central.

A particularly critical transitional phase in cognitive decline is Mild Cognitive Impairment (MCI), which bridges physiological and pathological aging (Petersen et al., 1997; Anderson, 2019; Lee, 2023). MCI is characterized by deficits in one or more cognitive domains (e.g., memory, attention, executive function, or language), greater than expected for the individual’s age and education level but, differently from a major neurocognitive disorder, do not significantly interfere with daily activities (Gauthier et al., 2006; APA, 2022). This condition represents a major clinical concern as it poses a risk of progression significantly higher compared to cognitively normal individuals, with annual conversion rates ranging from 10 to 15% (Bruscoli and Lovestone, 2004; Cooper et al., 2015; Anderson, 2019; Lee, 2023), reaching up to 60% within five years (Gauthier et al., 2006). Additionally, age-related cognitive decline in elderly adults is associated with decreased self-esteem, depression, and impaired ability to perform everyday activities, further increasing the risk of developing dementia (Artero et al., 2008; Avery et al., 2023; Munawar et al., 2023). Therefore, recognizing and addressing cognitive changes during the prodromal stage offers a crucial window for intervention, potentially delaying or reducing the likelihood of MCI-to-dementia progression or at least the severity of cognitive impairment (Manippa et al., 2024).

Several therapeutic approaches, including pharmacological treatments and cognitive rehabilitation programs, have been developed to manage cogntivive symptoms and preserve autonomy (Kasper et al., 2020; He et al., 2025). For instance, pharmacological interventions, including cholinesterase inhibitors (e.g., donepezil, galantamine, rivastigmine) and NMDA receptor antagonists (e.g., memantine), may provide modest improvements in cognitive functioning and daily activities, particularly in early disease stages (Chen, Y. et al., 2024; Wu and Fuh, 2025; Zolnowski-Kolp et al., 2025). Although beneficial, these medications are often complemented by compensatory interventions, including cognitive stimulation therapies, structured routines, environmental modifications, and caregiver support programs, all designed to optimize daily functioning and mitigate behavioral symptoms (Wu and Fuh, 2025; Lee et al., 2025).

In parallel, conventional cognitive training (CCT) has been widely implemented to enhance cognitive function through non-digital means, primarily focusing on task-specific training and manual-based intervention (Cicerone et al., 2011; Chen, J. W. et al., 2024). A central element of CCT is the repeated practice of activities that simulate everyday tasks and situations, tailored to each patient’s cognitive profile and daily life challenges. By targeting cognitive domains such as attention, executive functioning, memory, and problem-solving, CCT seeks to restore or compensate for impaired cognitive abilities (Cicerone et al., 2011; Chen, J. W. et al., 2024).

Despite their clinical relevance and wide application, these approaches come with several limitations. Pharmacological treatments may lose effectiveness as neurodegeneration progresses, while the repetitive nature of CCT tasks can make it difficult to sustain motivation and engagement over time, and limit opportunities for real-time, individualized feedback. Additionally, systematically tracking cognitive progress remains challenging, often relying on periodic assessments rather than continuous performance monitoring (Park et al., 2020), highlighting the need for more adaptive, engaging, and measurable rehabilitation strategies.

Therefore, there is a clear clinical need for interventions that are not only effective and measurable but also engaging for patients and capable of providing real-time feedback on cognitive performance. In the context of MCI, certain features of rehabilitation tools become particularly important. Among these, ecological validity, meaning the extent to which training resembles real-world activities, and adaptability to the individual’s cognitive level and progress are critical for promoting meaningful and transferable cognitive improvements. For instance, digital tools for remote early detection of MCI have introduced age-friendly serious games and mobile-based paradigms to assess cognitive functioning, hippocampal integrity (Colombo et al., 2024; Minta et al., 2026), as well as to identify individuals at increased risk for Alzheimer’s disease through personalized cognitive profiling (Coughlan et al., 2019). Recent advancements in information technology have led to the development of digital instruments not only for evaluation but also for intervention (Bonvino et al., 2025; Cornacchia et al., 2025; Cotelli et al., 2025).

Among these, Virtual Reality (VR)-based cognitive training (VRCT) represents a promising alternative to traditional interventions, offering immersive, interactive environments that simulate real-life scenarios, enhancing the ecological validity of cognitive exercises and allowing for adaptable, patient-tailored interventions (Liao et al., 2020; Zając-Lamparska et al., 2019). Unlike CCT, which largely relies on tabletop activities such as puzzles, wood blocks, and card games (Bahar-Fuchs et al., 2013; Fan et al., 2017; Kueider et al., 2012; Park, 2022), VR engages users through realistic, gamified tasks that promote motivation and active participation. Moreover, VR platforms can provide real-time performance feedback and adjust task difficulty, offering dynamic and personalized training experience. These features not only increase engagement but also improve the accessibility and cost-effectiveness of cognitive training programs (Liao et al., 2019). Although interest in VRCT as a potential ecological and effective intervention for MCI has grown, the current evidence base remains limited. A recent systematic review (Tortora et al., 2024) identified only three trials (130 participants), all suggesting that VRCT was at least as effective as CCT across cognitive and functional outcomes, with potential ecological and adaptive advantages. However, the review also highlighted the need for standardized protocols and more rigorous trials to clarify the long-term efficacy of VRCT and the role of immersion. At the same time, its scope was necessarily limited by the small number of available trials and by the heterogeneity of study designs. Moreover, VRCT effects were not systematically examined across specific cognitive domains, differences in immersion level, training intensity, or outcome variability were not addressed. It also lacked standardized effect size estimates and a detailed risk-of-bias assessment. These gaps underscore the need for an updated and methodologically rigorous synthesis focused exclusively on RCTs.

To address these gaps, this systematic review aims to update and critically evaluate the effectiveness of VRCT in individuals with MCI by comparing the effect of VRCT and CCT on cognitive and functional outcomes, focusing on randomized controlled trials (RCTs). By integrating effect size estimation, a domain-specific synthesis, and a systematic risk-of-bias assessment, this work provides a rigorous evaluation of current state-of-art. Based on preliminary evidence, we hypothesize that VRCT may yield greater pre-post cognitive improvements than CCT. At the same time, we expected substantial heterogeneity across studies and specific cognitive domains, due to differences in immersion level, training protocol, and outcome measures. Finally, it is important to acknowledge that the current evidence base remains limited by small sample sizes, short follow-up periods, and heterogeneous intervention protocols. These features characterize the field as a whole and inform the critical perspective adopted in this review. Through an updated and rigorous synthesis of evidence, this review seeks to clarify the clinical potential and current boundaries of VR-based cognitive rehabilitation in MCI, thereby informing practice, guide development of standardized protocols and support more effective and scalable cognitive rehabilitation strategies for aging populations.

## 2 Materials and methods

This systematic review aims to present and discuss RCTs comparing the effects of traditional interventions and VRCT for patients with neurodegenerative disorders. The methodological framework was designed in accordance with PRISMA (Preferred Reporting Items for Systematic Reviews and Meta-Analyses) guidelines to ensure comprehensive coverage and rigor in study selection (Moulaei et al., 2024); however, the review protocol was not registered in a public repository.

### 2.1 Search resources

**Figure 1:**
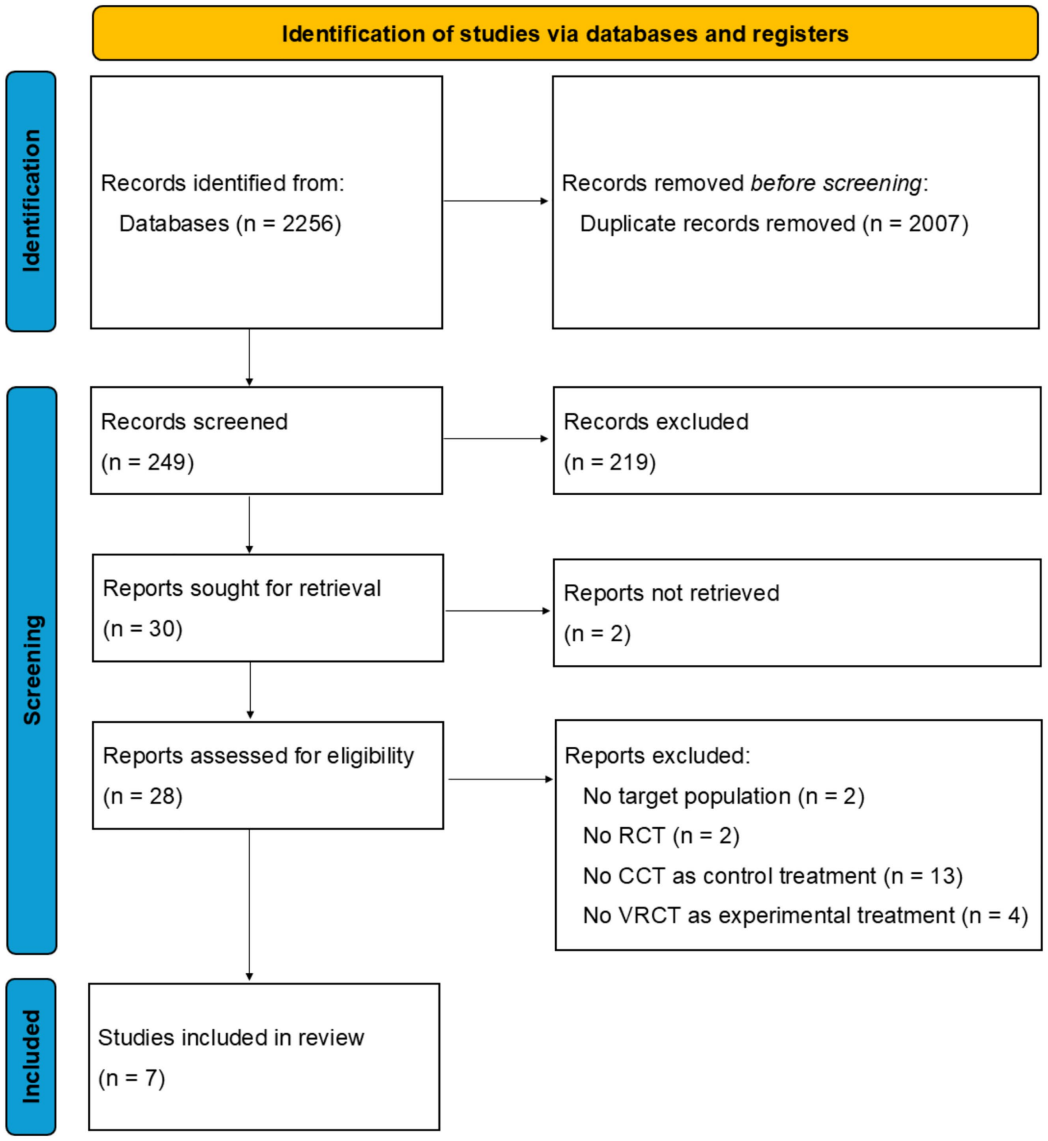
PRISMA flow diagram illustrates the systematic identification, screening, and inclusion of studies for review.

Two independent literature searches were conducted for article published until August 2025 to gather relevant studies using the three major scientific databases: Web of Science (WOS), Scopus and PubMed. The final search string selected to explore and interrogate the databases was (“virtual reality” OR “VR”) AND (“dementia” OR “Mild Cognitive Impairment” OR “MCI”). No time restrictions were applied, and only papers published in English were considered. The initial search yielded 2,256 articles. After removing duplicates, the study selection process followed a multi-step screening procedure. Three independent reviewers (GFS, VM, KHP) screened the remaining articles (*n* = 249) based on title and abstract. Full-text evaluations were then conducted on those assessed for eligibility (*n* = 28). After the disambiguation process, in which disagreements among the three reviewers were resolved through discussion, seven studies were included in the final review (Figure 1).

### 2.2 Eligibility criteria and study selection

No formal inter-rater reliability metrics (e.g., Cohen’s kappa) were calculated; however, systematic reconciliation was performed to reduce selection bias. Eligibility criteria were defined as *a priori* using a structured PICOS framework (Population, Intervention, Comparator, Outcomes, Study design), as detailed below.

*Population (P)* included older adults diagnosed with Mild Cognitive Impairment (MCI) due to neurodegenerative causes. Studies were excluded if they involved: only healthy older adults, patients with dementia of non-neurodegenerative etiology (e.g., vascular, traumatic), mixed samples without separable MCI data.

*Intervention (I)* consisted in Virtual Reality Cognitive Training (VRCT) delivered through immersive, semi-immersive, or non-immersive VR systems, either combined or not with other training (e.g., motor training). Studies focusing solely on feasibility, usability, or protocol development without clinical outcomes were excluded.

*Comparator (C)* was Conventional Cognitive Training (CCT) or other standardized, non-VR cognitive interventions. Studies were excluded if the control group received: no treatment, usual care, unspecified or heterogeneous activities not comparable to CCT.

*Outcomes (O)* included changes in cognitive functioning assessed through standardized neuropsychological instruments (i.e., primary outcomes), which can be mapped onto the DSM-5 cognitive domains for neurocognitive disorders (i.e., complex attention, executive function, learning and memory, language, perceptual-motor function, and general cognition), and functional autonomy and psychoaffective status (i.e., secondary outcomes). Studies were excluded if they did not report quantitative clinical outcomes (e.g., only feasibility or qualitative measures).

*Study design (S)* comprised randomized controlled trials (RCTs) only. Single-case studies, pilot or feasibility trials without a control arm, non-randomized or uncontrolled designs were excluded.

### 2.3 Data extraction and risk of bias assessment

A systematic collection of information from each of the selected studies, required for the analysis, comprised study design, sample characteristics (e.g., age, cognitive state, number of participants), specifics of the VRCT intervention (e.g., type of intervention, duration, frequency of interventions), cognitive functions assessed, and key results (Kim et al., 2019; Moreno et al., 2019). Discrepancies in data interpretation were addressed through group consensus. All extracted data are presented in Table 1. Due to the high heterogeneity in study designs, VRCT interventions, and outcome measures, a narrative synthesis approach was adopted (D’Cunha et al., 2019).

**Table 1:** Study design and methodology of the included RCTs.

Study	Design and sample	Type of interventions	Duration and frequency	Cognitive outcomes
Buele et al. (2024)	Thirty-four older adults with MCI assigned to two groups (VRCT *n* = 17 vs. CCT *n* = 17)	VRCT: motor training + VR-based cognitive training CCT: motor training + traditional cognitive training	20-min sessions twice a week over 6 weeks (12 sessions in total)	General Cognition (MoCA), Psychoaffective status (SGDS), and Functional Autonomies (iADL) at baseline and post-intervention
Liao et al. (2019)	Forty-two older adults with MCI assigned to two groups (VRCT *n* = 21, CCT *n* = 21)	VRCT: VR-based physical and cognitive training (semi-immersive) CCT: traditional physical and cognitive training	60-min sessions three times a week over 12 weeks (36 sessions in total)	Complex attention (TMT), Executive Functions (SCWT) at baseline and post-intervention
Liao et al. (2020)	Forty-two older adults with MCI assigned to two groups (VRCT *n* = 21, CCT *n* = 21)	VRCT: VR-based physical and cognitive training (semi-immersive) CCT: traditional physical and cognitive training	60-min sessions three times a week over 12 weeks (36 sessions in total)	General Cognition (MoCA), Executive Functions (EXIT-25), Learning and Memory (CVVLT), and Functional Autonomies (iADL) at baseline and post-intervention
Manenti et al. (2020)	Forty-nine older adults with MCI assigned to three groups (VRCT + hVCRT n = 18, VRCT + hCCT *n* = 14, CCT *n* = 17)	VRCT + hVRCT: Face-to-face cognitive VR treatment followed by home-based cognitive VR treatment VRCT + hUCT: Face-to-face VR treatment followed by home-based unstructured cognitive treatment CCT: Face-to-face cognitive group treatment	VRCT groups: 60-min sessions three times a week over 4 weeks (12 sessions) + 60-min sessions three times a week over 12 weeks (36 sessions) CCT: 60-min sessions three times a week over 4 weeks (12 sessions)	Learning and Memory (RAVLT, FCSRT), Language (Verbal fluency, Naming), Complex Attention (TMT), Perceptual-Motor Function (CDT) at baseline, post face-to-face intervention (1 month), post home-based intervention (4 months follow-up), and 7 months follow-up
Manenti et al. (2024)	One-hundred-nine older adults with MCI assigned to five groups (VRCT and atDCS + hVRCT *n* = 23, VRCT and ptDCS + hVRCT *n* = 21, VRCT + hVCRT *n* = 20, VRCT + hUCT *n* = 23, CCT *n* = 22)	VRCT and atDCS + hVRCT: Face-to-face cognitive VR treatment combined with active tDCS followed by home-based cognitive VR treatment VRCT and ptDCS + hVRCT: Face-to-face cognitive VR treatment combined with anodal tDCS of lDLPCF followed by home-based cognitive VR treatment VRCT + hVCRT, VRCT + hUCT and CCT groups received the same treatment as Manenti et al. (2020)	VRCT groups: 60-min sessions three times a week over 4 weeks (12 sessions) + 60-min sessions three times a week over 12 weeks (36 sessions) atDCS/ptDCA: 25 min of active anodal or placebo tDCS over DLPCF during face-to-face VR sessions CCT: 60-min sessions three times a week over 4 weeks	General Cognition (MMSE), Learning and Memory (RAVLT, FCSRT, ROCF Recall), Language (Verbal fluency, BADA), Perceptual-Motor Function (ROCF Copy), Executive Functions, Functional Autonomies (bADL, iADL), Psychoaffective Status (NPI, GDS) at baseline, post face-to-face intervention (1 month), post home-based intervention (4 months follow-up), and 7 months follow-up
Park et al. (2020)	Forty older adults with MCI assigned to two groups (VRCT *n* = 20, CCT *n* = 20)	VRCT: virtual cognitive-motor rehabilitation via MOTOcog system (non-immersive) CCT: tabletop conventional therapy	30-min sessions five times a week over 6 weeks (30 sessions in total)	General Cognition (MoCA), Complex Attention (TMT), Learning and Memory (DS) at baseline and post intervention
Torpil et al. (2021)	Sixty-four older adults with MCI assigned to two groups (VRCT *n* = 32, CCT *n* = 32)	VRCT: non-immersive computer-generated interactive environments in addition to CCT CCT: cognitive training based on LOTCA-G domains	45-min sessions twice a week over 12 weeks (24 sessions for each training)	General Cognition (LOTCA-G total score), Perceptual-Motor Function (Visual Perception, Spatial Perception, Motor praxis, Visuomotor LOTCA-G subscores), Executive Functions (Thinking Operation LOTCA-G subscore), Learning and Memory (Memory LOTCA-G subscore), Complex Attention (Attention/Concentration LOTCA-G subscore) at baseline and post intervention

VRCT = virtual reality-based cognitive training; CCT = conventional cognitive training; hVRCT = home-based virtual reality-based cognitive training hUCT = home-based unstructured cognitive training; VRCT + hVRCT = face-to-face VR-based cognitive training followed by home-based VRCT; VRCT + hUCT = face-to-face VR-based cognitive training followed by home-based unstructured cognitive training; MCI = mild cognitive impairment; ptDCS = placebo transcranial direct current stimulation; atDCS = active transcranial direct current stimulation; MoCA = Montreal cognitive assessment; SGDS: short-geriatric depression scale; iADL = instrumental activity of daily living; bADL: basic activity of daily living; NPI = neuropsychiatry inventory; SCWT = stroop color-word test; EXIT-25 = executive interview; CVVLT: Chinese version of verbal learning test; RAVLT = Rey auditory verbal learning test; FCSRT = free and cued selective reminding test; CDT = clock drawing test; ROCF = Rey–Osterrieth complex figure; DS = Digit Span; LOTCA-G = Loewenstein occupational therapy cognitive assessment-geriatric version. BADA = behavioral assessment of the dysexecutive syndrome.

The included studies were analyzed to detect recurring patterns and divergent findings, with a particular focus on general cognition and the five DSM-5 cognitive domains: complex attention, executive function, social cognition, language, perceptual-motor function, and learning and memory, with the latter further subdivided into immediate and delayed recall. In addition, psychoaffective status and functional autonomy were examined as functional outcomes. Comparative analyses between VRCT and CCT were performed to evaluate the therapeutic potential of VRCT in MCI care. When direct head-to-head comparisons were unavailable (all studies except Park et al., 2020), Cohen’s *d* was computed as a sample-based estimate, using the pooled standard deviation. For each cognitive and non-cognitive outcome, effect sizes were first derived from the within-subject change between baseline and post-intervention. These change scores were then compared between experimental and control groups to obtain between-group effect sizes Cohen’s *d*.


$$d=(\Delta M_{\text{VRCT}}-\Delta M_{\mathrm{CCT}})/{\mathrm{SD}}_{\text{pooled}}$$

$${\mathrm{SD}}_{\text{pooled}}=\surd \{[\begin{array}{l} (N_{\text{VRCT}}-1)\times {{\mathrm{SD}}_{\text{VRCT}}}^{2}\\ +(N_{\mathrm{CCT}}-1)\times {{\mathrm{SD}}_{\mathrm{CCT}}}^{2} \end{array}]/(N_{\text{VRCT}}+N_{\mathrm{CCT}}-2)\}$$

To account for potential small-sample bias, Hedges’ g was also computed; however, given the sample sizes of the included studies, the correction factor differed from 1 by less than 2%, resulting in values that were numerically indistinguishable from Cohen’s *d*. For clarity and consistency, Cohen’s *d* is therefore reported throughout the manuscript (see Table 2). Confidence intervals (95% CIs) for each effect size were calculated using the standard variance formula for between-group standardized mean differences, incorporating both sample sizes and the pooled standard deviation of the change scores. Because all included studies reported means and standard deviations for the change scores (post–baseline), confidence intervals were computed directly from group-level summary statistics. The standard error of Cohen’s *d* was calculated using the conventional formula for independent-group standardized mean differences:


$${\mathrm{SE}}_{d}=\surd \{\begin{array}{l} \left(N_{\text{VRCT}}+N_{\mathrm{CCT}})/(N_{\text{VRCT}}\times N_{\mathrm{CCT}}\right)\\ +d^{2}/[2\times (N_{\text{VRCT}}+N_{\mathrm{CCT}}-2)] \end{array}\}$$

**Table 2:** Summary of included study evaluating the effects of virtual reality cognitive training (VRCT) versus conventional cognitive training (CCT) on cognitive function, depressive symptoms, and activities of daily living.

Study	Main findings	Post-pre Cohen’s *d* (VRCT vs CCT)	Findings considerations
Buele et al. (2024)	General cognition (ps < 0.001) and depression (ps < 0.006) significantly improved after both interventions, while no significant group effect was found. No statistically significant improvement in ADL in either group.	MoCA = 0.04 SGDS = 0.55 (CCT > VRCT) iADL = 0.22 (VRCT > CCT)	Out of the 34 allocated patients, 26 completed the trial. No significant differences between VRCT and CCT intervention in any assessed outcome. Nevertheless, effect size estimation demonstrated that the CCT group reported a moderate yet uncertain reduction in depressive symptoms compared to the VRCT group. Small sample size and brief intervention duration limit the generalizability of the study.
Liao et al. (2019)	Significant improvements in executive function attention (SCWT Correct Response and Time) were found for both VRCT (*p* < 0.004) and CCT (*p* < 0.030). The VRCT group showed significant improvements in the TMT-B compared with CCT (*p* < 0.032).	TMT-A = −0.09, TMT-B = −0.49 (VRCT > CCT), Δ TMT = −0.52 (VRCT > CCT) SCWT Correct Response = 0.13 SCWT Time = −0.15	Out of the 42 allocated patients, 34 completed the trial. Outcome measures also included gait performance, with VRCT leading to greater improvements in cognitive dual-task gait performance. Effect size estimations demonstrate trivialsuperiority of VRCT in improving TMT-B and attentional shifting (Δ TMT) compared with CCT.
Liao et al. (2020)	Both groups improved in executive function and immediate recall. Only the VRCT group showed significant improvement in general cognition (*p* < 0.001), delayed recall (*p* = 0.002), and iADL (*p* < 0.001). The latter significantly improved after VRCT compared to CCT (*p* = 0.006).	MoCA = 0.38 (VRCT > CCT) EXIT-25 = −0.11 CVVLT Immediate Recall = 0.40 (VRCT > CCT) CVVLT Delayed Recall = 0.38 (VRCT > CCT) iADL = 0.52 (VRCT > CCT)	Out of the 42 allocated patients, 34 completed the trial. Outcome measures also included near-infrared spectroscopy (NIRS) over the prefrontal cortex, with the VRCT group showing decreased prefrontal activation after the intervention compared to the CCT. Effect size estimations demonstrate moderate superiority of VRCT in improving functional abilities (iADL). Lack of psychoaffective status screening and high dropout rate affect results reliability.
Manenti et al. (2020)	Regardless of the intervention, an improvement is observed in FCSRT IFR (*p* = 0.004) and DFR (*p* = 0.012), action naming (*p* = 0.005), and CDT (*p* = 0.001). No main group effect is found; however, significantly better performances in FCSRT IFR (*p* = 0.010), semantic verbal fluency (*p* = 0.024), and CDT (*p* = 0.010) were reported after VRCT compared to CCT.	RAVLT IR = −0.13, RAVLT DR = 0.10 FCSRT IFR = 0.37 (VRCT > CCT), FCSRT ITR = 0.23 (VRCT > CCT) FCSRT DFR = 0.18, FCSRT DTR = −0.02 FCSRT ISC = 0.26 (VRCT > CCT) Phonemic Verbal Fluency = −0.01 Semantic Verbal Fluency = 0.24 (VRCT > CCT) Object Naming = 0.11 Action Naming = −0.22 (CCT > VRCT) TMT-A = −0.3 (VRCT > CCT), TMT-B = −0.27 (VRCT > CCT) CDT = −0.47 (VRCT > CCT)	All the 49 patients included in the study completed the assigned treatments. Outcome measures also included the Everyday Memory Questionnaire and Quality of Life in AD scale, the former improving after both treatments. Effect size estimation demonstrates only a small yet uncertain superiority of VRCT in improving mostly memory and complex attention, while the improvement inperceptual-motor functions is more reliable. Study design also included hVRCT and hUCT following clinical treatments; however, their effects have not been further analysed as not pertinent to this review. Limited generalizability of the results is due to the small sample size.
Manenti et al. (2024)	Authors evaluated the efficacy of VRCT combined with either anodal or placebo tDCS and of CCT within the four timepoints. Hence, no statistical comparison between VRCT only and CCT was conducted.	MMSE = 0.32 (VRCT > CCT) bADL = 0.00 iADL = −0.18 RAVLT IR = 0.03, RAVLT DR = −0.07 FCSRT IFR = 0.24 (VRCT > CCT), FCSRT ITR = 0.13, FCSRT DFR = 0.04, FCSRT DTR = 0.02 FCSRT ISC = 0.52 (VRCT > CCT) ROCF Recall = 0.25 (VRCT > CCT) Phonemic Verbal Fluency = 0.01 Semantic Verbal Fluency = 0.34 (VRCT > CCT) Object Naming = −0.06 Action Naming = −0.35 (CCT > VRCT) ROCF Copy = 0.21 (VRCT > CCT) NPI = 0.03 GDS = −0.23 (VRCT > CCT)	Out of the 109 allocated patients, 105 completed the trial. Outcome measures also included the Everyday Memory Questionnaire and the Quality of Life in AD scale. The study design was similar to that of Manenti et al. (2020), with two additional groups receiving combined active vs. sham tDCS alongside VRCT + hVRCT or hUCT. Since the analysis focused on combined treatments, an improvement in episodic memory was observed following atDCS-VRCT, whereas no enhancement was reported after ptDCS-VRCT. Effect size estimation indicated a small yet uncertain superiority of VRCT in improving general cognition, memory performance, and perceptual-motor functions, while the small improvement in psychoaffective status in more reliable. However, these effects were not further analyzed, as they were not directly relevant to the scope of this review.
Park et al. (2020)	After the intervention, the VRCT group significantly improved in the MoCA (*p* = 0.045), TMT-A (*p* = 0.039), TMT-B (*p* = 0.040), and DS Forward (*p* = 0.011) compared to the CCT group.	MoCA = 0.80 (VRCT > CCT) TMT-A = −0.14, TMT-B = −0.60 (VRCT > CCT) DS Forward = 1.19 (VRCT > CCT), DS Backward = 0.20 (VRCT > CCT)	Out of the 40 allocated patients, 35 completed the trial. Effect size estimations demonstrate moderate superiority of VRCT in improving general cognition (MoCA), complex attention (TMT-B), and immediate recall (DS Forward) compared with CCT.
Torpil et al. (2021)	Both CCT and CCT + VRCT groups showed significant cognitive improvements (ps < 0.05). The CCT + VRCT group significantly improved in orientation, visual–spatial perception, visuomotor organization, thinking operation, and attention/concentration (*p* < 0.001). Praxis, however, improved more in the CCT group.	LOTCA-G Total Score = 1.48 (VRCT > CCT) LOTCA-G Visual Perception = 1.02 (VRCT > CCT) LOTCA-G Spatial Perception = 0 LOTCA-G Motor Praxis = 0.12 LOTCA-G Visuomotor = 1.47 (VRCT > CCT) LOTCA-G Thinking Operation = 0.39 (VRCT > CCT) LOTCA-G Memory = −0.07 LOTCA-G Attention/Concentration = 0.59 (VRCT > CCT) LOTCA-G Orientation = 0.44 (VRCT > CCT)	Out of the 64 allocated patients, 61 completed the trial. Effect size estimation demonstrates a moderate-to-strong superiority of combined CCT and VRCT compared with CCT only in improving the general score of LOTCA-G and specifically, visual perception, visuomotor, and attention/concentration subscores.

MoCA = Montreal cognitive assessment; SGDS: short-geriatric depression scale; iADL = instrumental activity of daily living; bADL: basic activity of daily living; TMT-A = trail making test part A; TMT-B = trail making test part B; Δ TMT = TMT B-A; SCWT: stroop color-word test; EXIT-25 = executive interview; CVVLT: Chinese version of verbal learning test; RAVLT IR = Rey auditory verbal learning test immediate recall; RAVLT DR = Rey auditory verbal learning test delayed recall; FCSRT IFR = free and cued selective reminding test immediate free recall; FCSRT ITR = immediate total recall; FCSRT DFR = delayed free recall; FCSRT DTR = delayed total recall; FCSRT ISC = index of sensitivity to cueing; CDT = clock drawing test; ROCF = Rey–Osterrieth complex figure; DS = digit span; LOTCA-G = Loewenstein occupational therapy cognitive assessment-geriatric version.

and the 95% confidence interval was obtained as:


$$\mathrm{CI}=d\pm 1.96\times {\mathrm{SE}}_{d}$$

**Figure 2:**
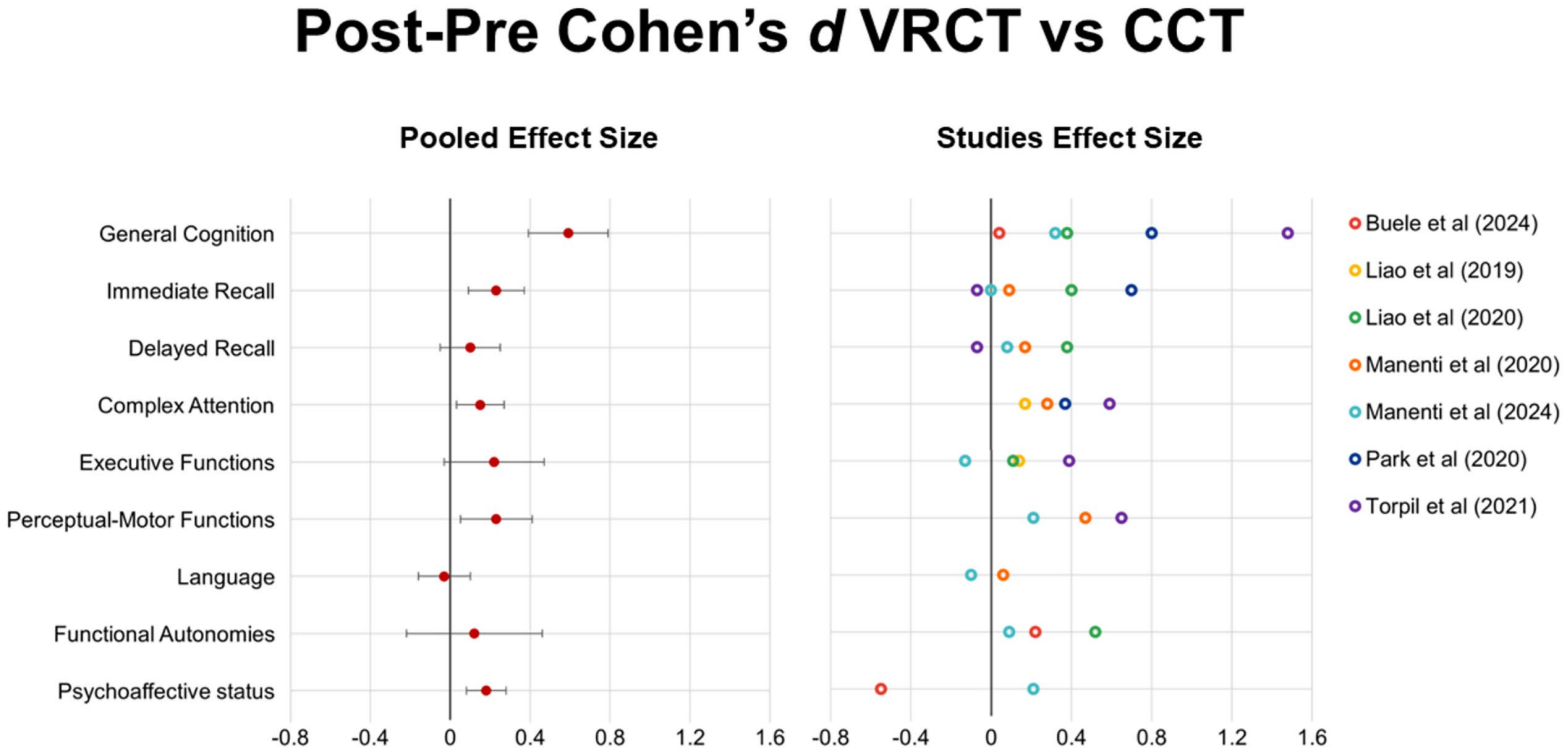
Post–pre effect sizes (Cohen’s *d*) for virtual reality cognitive training (VRCT) versus conventional cognitive training (CCT) across cognitive, affective, and functional domains. Positive values indicate greater improvement following VRCT, while negative values indicate greater improvement following CCT. For each cognitive domain, a pooled effect size was computed (red dots) by combining the available study-level Cohen’s d values using iverse-varaince weighting. Corresponding 95% confidence intervals reflect the variability of the pooled estimate within each domain. Colored points represents the effect size from each individual study.

These CIs quantify the precision of each estimate and are reported in the tables and visualized in Figure 2. This method provided a standardized framework for evaluating intervention effects across time points and outcome domains.

Finally, for each cognitive domain, a pooled effect size was calculated by combining the reported Cohen’s *d* values from the included studies. Standard errors were reconstructed from the 98% confidence intervals using the formula:


SE=(UpperCI−LowerCI)/(2×1.96)

Effect sizes were weighted by the inverse of their variance (𝑤_𝑖_=1/𝑆𝐸_𝑖_^2^) to compute a variance-weighted mean effect for each domain. Corresponding pooled 95% confidence intervals were derived accordingly. This procedure was applied to provide a quantitative summary of effects within the context of the present review.

The methodological quality of the included RCTs was assessed using the Cochrane Risk of Bias 2 tool (Sterne et al., 2019), evaluating potential bias across five domains: randomization process, deviations from intended interventions, missing outcome data, measurement of the outcome, and selection of the reported result. Signaling questions guided the judgments within each domain, leading to an overall rating of low risk, some concerns, or high risk of bias. Three authors (VM, GFS, MG) conducted the assessment independently, and disagreements were resolved through discussion. When available, study protocols or trial registrations were used to check whether outcomes and analysis plans had been pre-specified.

## 3 Results

**Figure 3:**
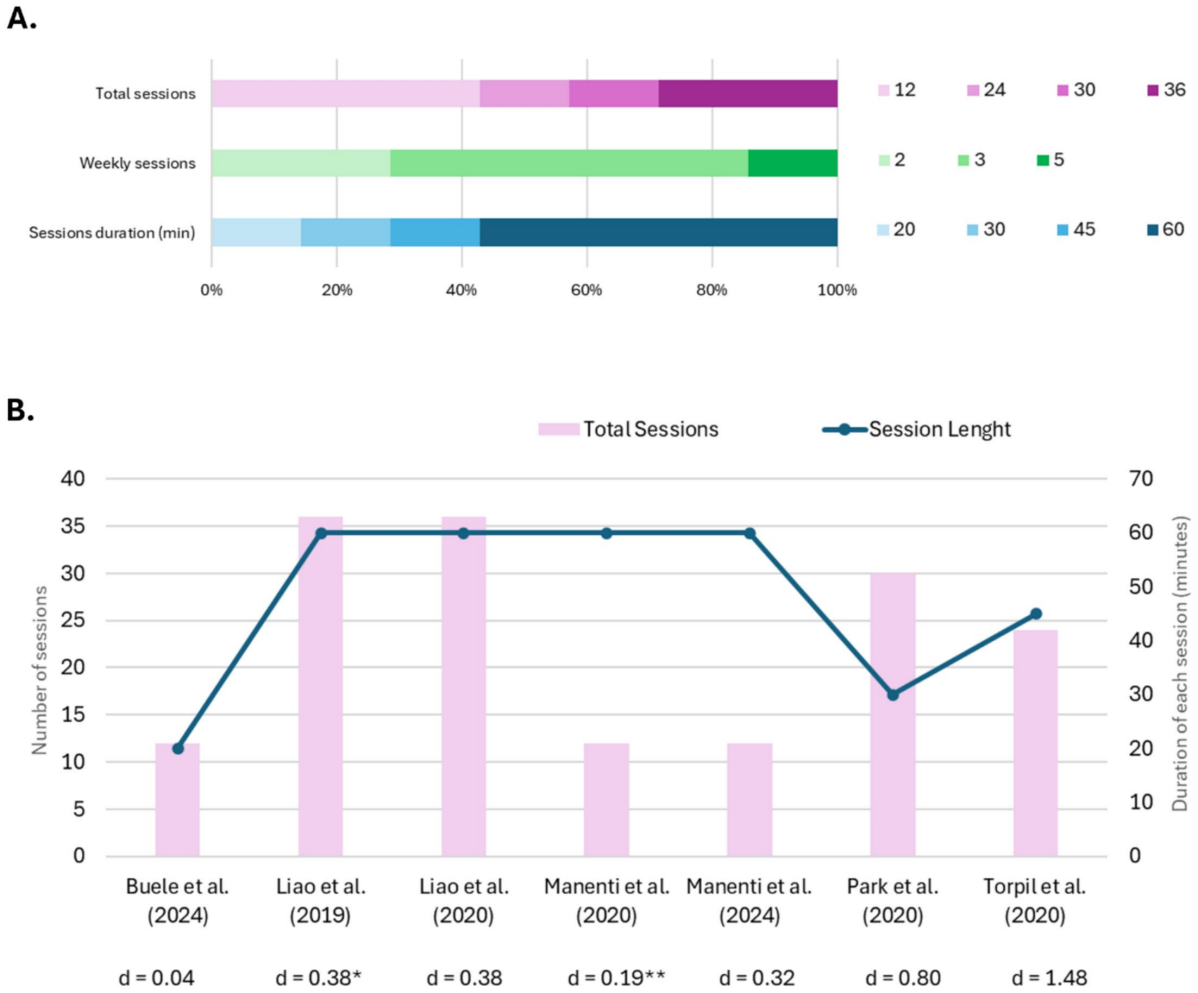
Protocols and study characteristics. **(A)** Distribution of training protocols across studies, showing the proportion of protocols using different total number of sessions (12, 24, 30, or 36), weekly frequency (2, 3, or 5 sessions per week), and session duration (20, 30, 45, or 60 min). **(B)** Comparison of intervention parameters across included studies, showing the total number of sessions (pink bars, left y-axis) and session duration (blue line, right y-axis). Effect sizes (Cohen’s *d*) for general cognition performance are reported below each study reference (positive values indicate VRCT advantage over CCT). Although not presented in the reported outcomes, for descriptive purposes, * Liao et al. (2019) effect size was obtained by Liao et al. (2020) while ** Manenti et al. (2020) *d-*value was computed by averaging all the other cognitive domain effect sizes.

Seven RCTs involving 380 older adults with MCI (348 analyzed) were included in this review and summarized in Table 1. Sample sizes ranged from 34 to 109 participants per trial. Five studies employed immersive or semi-immersive VR systems (head-mounted displays or projection-based setups), while two operated non-immersive desktop-based or motion-capture platforms (Park et al., 2020; Torpil et al., 2021). Interventions were structured as multi-week programs, administered 2-to-5 times per week, each session lasting between 20 and 60 min, amounting to a total of 12 to 36 sessions across each trial. Outcomes assessed encompassed general cognition (i.e., MoCA, MMSE, LOTCA-G), learning and memory (i.e., CVVLT, RAVLT, FCSRT, and Digit Span), executive function (i.e., SCWT, EXIT-25), complex attention (i.e., TMT), perceptual-motor function (i.e., CDT, ROCF), language (i.e., Verbal Fluency, Naming Test), functional autonomy (i.e., iADL, bADL), and psychoaffective status (i.e., GDS, SGDS, NPI). The social cognition domain was not assessed in any of the studies included. A summary of protocol designs and general cognition effect size are reported in Figure 3.

### 3.1 Interventions effect sizes

All the effects of CCT and VRCT on cognitive and functional domains are summarized in Table 2 and Figure 2. Across cognitive and functional domains, the most robust and consistent effects of VRCT compared to CCT emerged in general cognition, which showed a moderate average effect size (*d* = 0.59; 95% CI = 0.39, 0.79). Small but consistent positive effects were observed for immediate recall (*d* = 0.23; 95% CI = 0.09, 0.37), perceptual-motor functions (*d* = 0.23; 95% CI = 0.05, 0.41), complex attention (*d* = 0.15; 95% CI = 0.03, 0.27), and psychoaffective status (*d* = 0.18; 95% CI = 0.08, 0.28), with confidence ranges largely favoring VRCT. Executive functions, delayed recall, language and functional autonomies showed small or null benefits with substantial heterogeneity across studies.

In the multicenter, randomized, active-controlled trial of Manenti et al. (2020), 49 participants with MCI were recruited and assigned to 3 groups: (a) 18 participants received 12 sessions of face-to-face VRCT over 4 weeks, followed by 36 sessions of home-based VRCT, three sessions per week (VRCT + hVRCT); (b) 14 received face-to-face VRCT followed by 36 sessions of at-home unstructured cognitive treatment, three sessions per week (VRCT + hUCT); and (c) 17 participants received 12 sessions of face-to-face group CCT. Cognitive (i.e., memory, language, attentional functions, and visuo-constructional abilities) and functional assessments were conducted at baseline, post face-to-face intervention (1 month), post at-home intervention (4 months), and 7 months after baseline. Authors generally reported improvements in memory, language, and visuo-constructional abilities after VRCT treatments compared to CCT, suggesting that VRCT + hVRCT induces more maintenance of the obtained gains than VRCT + hUCT (T2 and T3). We estimated the effect size only after face-to-face interventions by collapsing the two VRCT groups. VRCT interventions compared to CCT yielded small improvements in the immediate free (IFR, *d* = 0.37) and total recall (ITR, *d* = 0.23) of the Free and Cued Selective Reminding Test (FCSRT) and for the Index of Sensitivity to Cueing (ISC, *d* = 0.26). In the language domain, VRCT participants showed small benefits in semantic fluency (*d* = 0.24) compared with CCT, while a negative effect was found for action naming (*d* = −0.22). Attentional measures indicated modest reductions in completion times at the Trail Making Test (TMT) after the VRCT (TMT-A *d* = −0.30; TMT-B, *d* = −0.27). Visuoconstructional performance, assessed by the Clock Drawing Test (CDT), followed a similar trend (*d* = −0.47).

Adopting a similar study design, the same authors (Manenti et al., 2024) randomly assigned 109 MCI elderly to 5 different groups: (a) VRCT with anodal transcranial direct current stimulation (tDCS) followed by Home VRCT, (b) VRCT with sham tDCS + Home VRCT, (c) VRCT + Home VRCT (VRCT + hVRCT), (d) VRCT + home unstructured stimulation (VRCT + hUCT), or (e) group cognitive stimulation (CCT). All the participants enrolled in the study received twelve 60-min sessions of face-to-face training over 4 weeks. A comprehensive cognitive and functional assessment was conducted at baseline, post face-to-face interventions, post at-home intervention, and 7 months after the intervention concluded. To guarantee a consistent comparison with the other selected studies in this review, CCT post face-to-face intervention effects were compared only to VRCT groups (collapsing VRCT + hVRCT and VRCT + hUCT participants). A modest improvement in general cognition score (assessed through MMSE) and depression symptoms (GDS) was found in VRCT groups compared to CCT at post-intervention (*d* = 0.32, *d* = −0.23, respectively). Concerning memory outcomes, VRCT participants showed modest gains in Rey-Osterrieth Complex Figure (ROCF) delayed recall (*d* = 0.25) and IFR (*d* = 0.24) and moderate improvement in ISC (*d* = 0.52). Visuoconstructional performance, assessed by the ROCF copy, followed a similar trend (*d* = 0.31). In the language domain, VRCT participants showed small benefits in semantic fluency (*d* = 0.34) compared with CCT, while a negative effect was found for action naming (*d* = −0.35).

In Buele et al. (2024), a single-blind RCT enrolled 34 community-dwelling older adults with MCI (MoCA-S 19–25) and randomized them to 6 weeks of bi-weekly motor training combined with either immersive VR-based (VRCT, *n* = 17) or pencil-and-paper (CCT, *n* = 17) cognitive tasks. General cognition (as measured by the Montreal Cognitive Assessment, MoCA), depressive symptoms (assessed using the short form of the Geriatric Depression Scale, SGDS), and functional autonomies (evaluated through Instrumental Activities of Daily Living, iADL) were assessed immediately before and after the intervention. Between-group comparisons showed no significant differences in MoCA, SGD, or iADL. Both groups exhibited large intragroup gains in general cognition (CCT: *p* < 0.001, VRCT: *p* < 0.001) and mood (CCT: *p* < 0.001, VRCT: *p* = 0.005), while neither group showed significant improvements in daily-living functions. Between-group effect size comparisons revealed a significantly greater improvement in depressive symptoms in the CCT compared with the VRCT group (*d* = 0.55) but not in general cognition.

Park et al. (2020) conducted an RCT assigning community-dwelling older adults with MCI to either VRCT (*n* = 18) or CCT (*n* = 17), both delivered over 6 weeks (30 min per day, 5 days per week). General cognition, assessed through MoCA, significantly improved in the VRCT group compared to the CCT (*d* = 0.8; *p* = <0.001). Concerning the TMT performance, although a smaller improvement (*d* = 0.14; *p* = <0.001) was observed in processing speed (TMT-A), a greater gain was measured in attentional shifting (TMT-B; *d* = 0.60, *p* = 0.009). Working memory outcomes yielded mixed results instead, as digit span forward performance improved significantly with a large effect size (*d* = 1.19, *p* < 0.001), while backward digit span gains were modest and not statistically significant (*d* = 0.20, *p* = 0.468).

In the single-blind RCT by Liao et al. (2019, 2020), 34 community-dwelling older adults with MCI were assigned to either VR-based physical and cognitive training (VRCT; *n* = 18) or conventional physical-cognitive training (CCT; *n* = 16). Their outcomes, assessed at baseline and post-intervention, were reported in two separate papers. In the first one (2019) the authors included executive functions evaluation (TMT and Stroop Color-Word Test; SCWT), showing that VRCT determined greater improvements in attentional shifting performance (TMT-B, *d* = −0.49; ΔTMT, *d* = −0.52), while negligible changes were observed in the SWCT performance. Here the authors also evaluate gait outcomes. In the second publication (Liao et al., 2020) outcomes spanned general cognition, executive function, episodic memory, and functional status. VRCT participants demonstrated small improvements in general cognition (MoCA; *d* = 0.38) and in verbal episodic memory (RAVLT), with comparable gains for both immediate (*d* = 0.40) and delayed recall (*d* = 0.38). In contrast, executive function showed negligible group differences on the Executive Interview-25 (EXIT-25). Importantly, VRCT was associated with a moderate benefit in functional autonomies (iADL, *d* = 0.52).

In Torpil et al. (2021) RCT, 61 older adults with MCI were assigned to two groups: 31 participants received standard cognitive rehabilitation (CCT), and 30 participants received cognitive rehabilitation enhanced with virtual reality (CCT + VRCT). Both interventions lasted 12 weeks. Cognitive functions were assessed using the Loewenstein Occupational Therapy Cognitive Assessment - Geriatric Version (LOTCA-G) battery before and after the intervention. Both groups demonstrated significant within-group improvements across multiple cognitive domains. However, between-group effect size comparisons indicated that the CCT + VRCT group achieved superior gains in visuomotor organization (*d* = 1.47), visual perception (*d* = 1.02), and the LOTCA-G total score (*d* = 1.48), while moderate benefits were also observed for attention and concentration (*d* = 0.59), orientation (*d* = 0.44), and thinking operations (*d* = 0.39). In contrast, spatial perception, memory, and motor praxis scores showed minimal-to-negligible differences between groups.

Overall, findings indicate that VRCT yields its strongest and most consistent advantages in global cognitive functioning, with smaller and more domain-specific benefits in attention, memory encoding, and perceptual-motor performance.

### 3.2 Risk of Bias assessment

**Figure 4:**
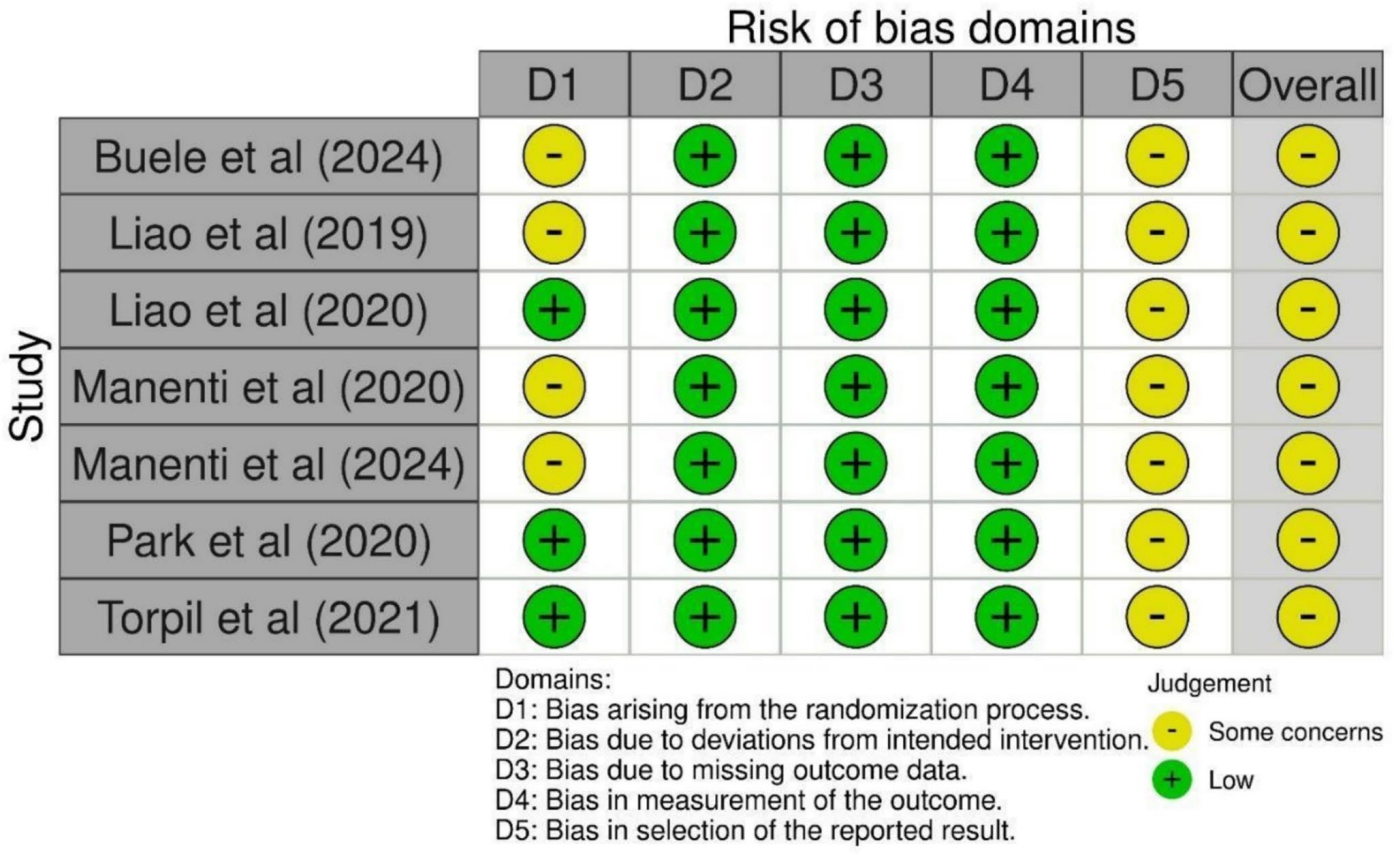
Risk of bias assessment across included studies using the Cochrane RoB 2 tool.

The risk of bias assessment using RoB 2 revealed that none of the included trials achieved an overall rating of low risk of bias (Figure 4). A frequent source of concern was related to the randomization process (Domain 1). While all studies reported using random allocation, concealment was sometimes insufficiently described. Moreover, doubts on the success of randomization were raised due to baseline imbalances between groups. As a result, four of the seven trials (Buele et al., 2024; Liao et al., 2019; Manenti et al., 2020, 2024) were judged as having some concerns in this domain, while the remaining three were rated at low risk.

In contrast, all trials were judged at low risk of bias for deviations from intended interventions (Domain 2), missing outcome data (Domain 3), and measurement of the outcome (Domain 4). Finally, the selection of the reported result (Domain 5) consistently raised some concerns across all studies. Trial registrations and protocols did not include sufficient information on clear analysis plans, and in two cases (Park et al., 2020; Torpil et al., 2021), their absence raised particular uncertainty. Although selective reporting of outcomes was not evident, the lack of prespecified analyses meant that this domain could not be rated at low risk.

The RoB 2 assessment indicated that all seven studies were evaluated, having some concerns regarding the overall risk of bias, primarily due to limitations in the reporting of randomization procedures and the lack of pre-specified analysis plans.

## 4 Discussion

This systematic review synthesized evidence from seven RCTs comparing the effects of VR-based cognitive training (VRCT) and conventional cognitive training (CCT) in older adults with MCI. According to the included studies, sample sizes ranged from 34 to 64 participants, except for one multicenter trial enrolling 109 individuals. VR interventions were predominantly non-immersive or semi-immersive, often integrating motor training. Typical intervention consisted of 60 min sessions, administered three times per week, for a total of 12 to 36 sessions over 4–12 weeks. Despite variability in intervention designs and outcome measures, the overall findings suggest that VRCT improves cognitive functioning better than CCT, mainly in complex attention and general cognition. Some trials also demonstrated positive effects on other cognitive domains and functional autonomy (IADL, BADL), although the latter were less consistently evaluated.

Across the seven RCTs, evidence consistently favors VRCT over CCT, although the magnitude of benefit varies widely across studies and domains. As shown in Figure 3, only two trials demonstrated clear VRCT superiority (*d* > 0.5): (Torpil et al., 2021) yielded large effects on perceptual-motor function, complex attention, and general cognition, assessed through LOTCA-G after two 45-min weekly sessions over 12 weeks. The strong alignment between the cognitive exercises and the LOTCA-G battery likely affected sensitivity, though it also demonstrates the potential of tightly targeted interventions. Moreover, the VRCT also received CCT, hence cognitive improvement was due to a combined rather than a single intervention. Park et al. (2020) also reported strong gains in immediate recall and general cognition (assessed through Digit Span and MoCA, respectively) after 30 high-frequency sessions (5 30-min weekly sessions). Similar to the previous study, this protocol involved a combined intervention of VRCT with motor training, while the CCT group underwent tabletop conventional therapy, suggesting that training intensity and integration of motor-cognitive tasks affected outcomes. Although results were not as strong, both studies also highlighted VRCT superiority compared to CCT in other cognitive domains. Two other studies published by Liao et al. (2019, 2020) reported a moderate advantage of VRCT over CCT. In particular, the authors conducted a RCTs that examined different outcomes, demonstrating that VRCT produced modest improvements in complex attention (TMT), learning and memory (Chinese Version of Verbal Learning Task; CVVLT), general cognition (MoCA), and functional autonomy (iADL) compared with CCT. Both groups followed a combined motor-cognitive intervention protocol. In addition, (Liao et al., 2019) assessed motor functioning and found no differences between the two interventions; however, these outcomes were not further analyzed. Their protocol consisted of 60-min sessions administered three times a week over 12 weeks (36 sessions in total).

Other studies showed weaker advantage of VRCT over CCT, like those conducted by Manenti et al. (2020, 2024). Overall, these two works came with a complex study design. The first one (Manenti et al., 2020) included at-home interventions (unstructured cognitive or VR-based) administered after the face-to-face VRCT, while the second study (Manenti et al., 2024) included two more groups undergoing active or placebo tDCS combined with VRCT. Finally, the authors included 3 and 7-month follow-up assessments. They reported that VRCT, followed by home-based VRCT or combined with active tDCS, appears to promote greater maintenance of the achieved gains compared to unstructured home-based stimulation or sham tDCS. However, to compare Manenti’s results with those of the other RCTs, we focused on the post-intervention effect sizes of face-to-face VRCT vs. CCT. Despite the comprehensive assessment provided, only a small advantage of the VRCT was found in the 2020 study for learning and memory (FCSRT), complex attention (TMT), and perceptual-motor function (CDT). Moreover, the 2024 outcomes yielded only a small improvement in general cognition (MMSE) after VCRT compared to CCT, while the improvements in the language domain were unclear since the former determined an improvement in semantic verbal fluency, while the latter favored action naming. Finally, in their RCT, Buele et al. (2024) found that depressive symptoms (SGDS) improved more after CCT than VRCT, although VRCT determined a small advantage for functional autonomies (iADL), while no differences were observed for general cognition. Both VRCT and CCT interventions were combined with motor training and consisted of short (20 min) low-intensity six-week protocol.

Concerning the cognitive measures and domains assessed, general cognition showed consistent improvements, ranging from small effects in Buele et al. (2024) RCT (MoCA *d* = 0.04) to large effects in Park et al. (2020) (MoCA *d* = 0.80) and Torpil et al. (2021) (LOTCA-G total *d* = 1.48). The stronger effects in the former study likely reflect the high training intensity (30 sessions over 6 weeks), while the second study reflects the effects of a cognitive intervention strongly tailored to the outcome measures (LOTCA-G). Accordingly, the same authors also reported the stronger benefit due to VRCT compared with CCT in perceptual-motor function, complex attention, and immediate recall. In contrast, the smaller effects in Buele RCT may be explained by the limited duration of the intervention (only twelve 20-min sessions).

Although the learning and memory domain has been extensively assessed, the results yielded only small-to-moderate effects favoring VRCT. Similar findings were reported for complex attention, executive functions, and perceptual-motor functioning. Only two studies examined the language domain, yet reported comparable effects between interventions (Manenti et al., 2020, 2024). Social cognition, instead, was the only cognitive domain not assessed by any of the studies included. On the other hand, VRCT demonstrated weak-to-inconsistent effects on psychoaffective status (mostly depressive symptoms) and functional autonomies compared to CCT, although being assessed only by three RCTs (Buele et al., 2024; Liao et al., 2020; Manenti et al., 2024).

## 5 Conclusions, limitations and future directions

The present findings suggest that VRCT may be a complementary rehabilitation approach for older adults with MCI, particularly for targeting complex attention and general cognitive functioning. Through adaptive and ecologically valid training environments, VRCT may be implemented in structured rehabilitation programs to enhance engagement and personalize intervention intensity. Clinicians should therefore consider VRCT as an adjunctive intervention implemented within multidisciplinary rehabilitation programs, while carefully monitoring patient response and feasibility. Further evidence is required to define optimal treatment parameters, long-term effectiveness, and cost-effectiveness.

An important limitation of the present review concerns the absence of prospective protocol registration. Although the review followed PRISMA guidelines, the protocol was not registered in a public repository. Prospective registration improves transparency and helps minimize potential reporting bias; therefore, future updates of this review will aim to include protocol registration. A major limitation across the included RCTs was the small sample size, as most trials were conducted with relatively small cohorts, typically ranging from 30 to 61 participants, while only one multicenter trial recruited over 100 individuals divided into 5 groups (Manenti et al., 2024). Such limited sample sizes reduce statistical power, increase the risk of type II errors, and restrict the generalizability of the findings to the broader population of individuals with MCI or dementia. Future research may benefit from multicenter collaborations and shared research infrastructures to increase recruitment capacity; standardized VR platforms and shared intervention protocols across institutions could reduce development costs and enhance comparability. In addition, portable or home-based VR systems may facilitate broader recruitment and improve accessibility for older adults with mobility limitations. Furthermore, high heterogeneity in protocol design (e.g., length and number of sessions, combined interventions) impairs comparisons and general conclusions on the effectiveness of VRCT. Finally, data-sharing initiatives and coordinated trial networks could further support large-scale randomized studies capable of producing more generalizable evidence. Another important limitation concerns the lack of follow-ups in most studies, as it does not allow comparison of the sustained VRCT and CCT effects and the long-term efficacy of the interventions. Incorporating longer follow-up periods would help to more accurately determine the enduring impact of these interventions. An additional weakness of the included studies is the variability in outcome measures. Different trials employed diverse cognitive and functional assessments, ranging from general cognition tests to specific cognitive domain measures. Only few studies included additional outcomes like quality of life or psychiatric symptoms and most studies did not employ neuroimaging techniques, making it difficult to ascertain whether the observed cognitive improvements were underpinned by corresponding neural changes. Although one study (Liao et al., 2020) incorporated NIRS, it was restricted to the prefrontal cortex, thereby providing limited spatial coverage and potentially overlooking training-related effects in other regions, such as the temporal and parietal cortices, critical in memory and executive functioning.

Despite these limitations, this review highlights the strong potential of VRCT as a modern tool for cognitive rehabilitation in dementia care. To address current methodological heterogeneity, future studies should follow more standardized protocols to enhance comparability and strengthen the outcomes. First, interventions should clearly report immersion level, task characteristics, training intensity, and duration; longer sessions administered multiple times a week may represent a reasonable starting point for future trials. Studies should also include a set of standardized outcome measures covering general cognition, domain-specific performance, functional autonomy, and psychoaffective outcomes. Follow-up assessments are recommended to evaluate sustained effects. Most importantly, trials should incorporate transparent reporting of allocation procedures, blinding, adherence rates, and adverse events to reduce the risk of bias. Finally, integrating neuroimaging or neurophysiological measures alongside behavioral outcomes may help clarify the neural mechanisms underlying training-related improvements. Together, these guidelines could help solidify VRCT as an effective and personalized approach to cognitive rehabilitation for individuals with neurodegenerative conditions, and enable reliable metanalyses to identify optimal parameters and compare the most effective protocols.

## Data Availability

Data and code sharing is not applicable to this article as no datasets were generated or analyzed during the current study.
